# Short- and Mid-Term Outcomes of Fixed-Dose Tramadol/Paracetamol in Early-Stage Symptomatic Knee Osteoarthritis: A Single-Center Retrospective Observational Extension Study

**DOI:** 10.3390/life16060962

**Published:** 2026-06-08

**Authors:** Valerio Cipolloni, Marco Bonifacio, Marco Giuseppe Musorrofiti, Raoul Saggini, Alessia Caldarola, Gilberto Grossi, Roberto Piazza, Mario Mangrella, Deborah Trastulli, Alessandro Conforti

**Affiliations:** 1 Azienda Ospedaliera Universitaria Università degli Studi della Campania Luigi Vanvitelli, 80100 Naples, Italy; valeriocipolloni@gmail.com; 2 Fisioterapia Medica srl, 00053 Civitavecchia, Italy; marcobonifaicio1969@gmail.com; 3 Department of Ergonomics, eCampus University, 22060 Novedrate, Italy; 4 Faculty of Psychology, eCampus University, 22060 Novedrate, Italy; 5 U.O.C. Riabilitazione, Villa dei Pini, 00042 Anzio, Italy; 6 Neurochirgia, Ospedale Israelitico, 00148 Rome, Italy; 7 Italfarmaco (Italy), 20216 Milan, Italy; r.piazza@italfarmacogroup.com (R.P.);; 8 Rheumatology, Helth Care asl roma 4, 0053 Civitavecchia, Italy

**Keywords:** osteoarthritis, tramadol, paracetamol, fixed-dose combination, WOMAC, numeric rating scale, Pittsburgh sleep quality index, retrospective observational study, real-world outcomes

## Abstract

**Background:** A previous short-term retrospective analysis from our center suggested early improvement in pain, function, and sleep outcomes after fixed-dose tramadol/paracetamol therapy in patients with early-stage symptomatic knee osteoarthritis (KOA). However, the durability of these outcomes beyond the initial treatment phase remains insufficiently described in routine clinical practice. **Objective:** To describe short- and mid-term patient-reported outcomes and safety during extended follow-up in adults with symptomatic KOA treated with fixed-dose tramadol/paracetamol in routine outpatient care. **Methods:** This single-center retrospective observational extension study analyzed a fixed cohort of 30 adults with symptomatic knee osteoarthritis and Kellgren–Lawrence grade I–II treated with fixed-dose tramadol/paracetamol in routine outpatient care. Patients were evaluated at baseline (T0), 15 days (D15), 3 months (M3), and 6 months (M6), where evaluable follow-up data were available. The primary outcome was change in pain intensity measured by the Numeric Rating Scale (NRS). Secondary outcomes included WOMAC total and subscale scores, Pittsburgh Sleep Quality Index (PSQI) score, adverse events, and treatment discontinuation. **Results:** Patients had a mean age of 64.8 ± 9.6 years and a mean BMI of 27.3 ± 5.0 kg/m^2^. NRS pain improved from a median of 6.0 (5.0–7.0) at baseline to 4.0 (2.0–5.3) at D15 and 3.0 (2.0–5.0) at both M3 and M6. WOMAC total score improved from 47.8 ± 12.8 at baseline to 35.6 ± 13.8 at D15, 33.8 ± 13.6 at M3, and 33.9 ± 13.5 at M6. Sleep score improved from 9.5 (7.0–15.0) at baseline to 7.0 (5.0–9.0) at D15 and remained improved at M3 and M6. Mild adverse events were reported in 10.0% of patients, and discontinuation occurred in 6.7%. **Conclusions:** In this small, uncontrolled, single-center retrospective cohort of adults with early-stage symptomatic KOA, fixed-dose tramadol/paracetamol was associated with early improvement in pain, WOMAC outcomes, and sleep, with group-level benefits remaining evident through 6 months. These findings should be considered exploratory and hypothesis-generating rather than comparative evidence of effectiveness. Larger controlled studies are needed to confirm durability, comparative benefit, and long-term safety.

## 1. Introduction

Osteoarthritis (OA) is a highly prevalent, progressive musculoskeletal disorder and a major cause of chronic pain, disability, and reduced quality of life in aging populations. It commonly affects weight-bearing joints, especially the knee and hip, where persistent pain and movement limitation compromise daily function and independence. Beyond pain alone, OA has broader clinical consequences, including sleep disturbance, reduced mobility, psychosocial stress, and increased healthcare utilization. Real-world evidence shows that OA and other musculoskeletal disorders contribute substantially to global disability, and that this burden is expected to increase further as populations age [1]. Pharmacological management of OA pain remains challenging because available analgesic strategies involve trade-offs between efficacy, tolerability, and patient-level safety risks. Network meta-analytic evidence suggests that some oral non-steroidal anti-inflammatory drugs (NSAIDs) provide meaningful analgesic benefit, but this may be offset by increased adverse events or treatment discontinuation in higher-risk populations [2]. Opioid-class therapies, although frequently prescribed, appear to offer limited average clinical benefit relative to their harm profile, with substantially higher risks of adverse events and withdrawal due to intolerance [2]. These considerations highlight the need for pragmatic analgesic strategies that may provide symptom control while limiting cumulative toxicity and treatment burden.

In this context, combination analgesics have been proposed as a rational multimodal approach to pain modulation. Combining agents with different mechanisms may improve pain relief at lower doses of individual components, potentially reducing dose-dependent toxicity while targeting both nociceptive and centrally mediated pain processes. Reviews of fixed-dose combination therapy emphasize that monomodal analgesia may be insufficient for clinically significant pain states and that multi-mechanistic combinations may offer additive or synergistic benefit [3]. For OA, however, such strategies must be evaluated not only in terms of pain reduction, but also with regard to function, sleep, treatment persistence, and tolerability over time.

The fixed-dose tramadol/paracetamol combination is a commonly used option in real-world musculoskeletal pain management. Tramadol is an atypical opioid with mixed mechanisms, whereas paracetamol remains one of the most frequently used early pharmacologic therapies in OA and lower back pain, often as part of combination regimens in routine care [1]. In clinical practice, such regimens may be considered when symptoms remain insufficiently controlled, particularly in patients who cannot tolerate NSAIDs or in whom NSAID use is limited by comorbidity burden. Older adults with OA frequently present with multimorbidity, polypharmacy, and altered drug sensitivity, all of which complicate analgesic decision-making and magnify safety concerns [1]. At the same time, the role of tramadol in OA remains controversial. The updated Cochrane review found that tramadol, alone or in combination with acetaminophen, produced no important mean benefit in pain or function compared with the placebo across randomized trials, although a slightly greater proportion of patients achieved clinically important improvement [4]. However, tramadol was also associated with higher rates of adverse events and a substantially increased likelihood of withdrawal due to intolerance [4]. These findings indicate that tramadol-based therapy should not be evaluated solely by mean pain reduction, but rather by its broader balance of symptom relief, tolerability, discontinuation, and patient-centered outcomes.

Recent evidence suggests that paracetamol-based combinations may still provide meaningful short- to intermediate-term benefit in selected OA populations. A systematic review and meta-analysis reported that paracetamol plus oral tramadol reduced pain compared with the placebo at intermediate follow-up in OA, with moderate-certainty evidence for pain reduction, while also noting heterogeneity across studies and the limited availability of long-term data [5]. This is particularly relevant because OA management often takes place under conditions of limited long-duration randomized evidence and substantial patient variability. Adding to this evidence base, a recent retrospective observational study evaluated tramadol/paracetamol 75 mg/650 mg in early-stage knee OA (Kellgren-Lawrence grade I–II) and found clinically meaningful short-term improvement in pain, function, and sleep quality over 15 days, with relatively low rates of adverse events and no treatment discontinuation [6]. Although the design and sample size limited causal inference, these findings suggested that in selected patients, particularly those with earlier-stage disease, tramadol/paracetamol may offer practical short-term symptomatic relief with acceptable tolerability [6].

Despite the real-world use of tramadol/paracetamol and other paracetamol-based combinations, important evidence gaps remain regarding the durability of benefit and the pattern of tolerability beyond the initial treatment phase. Systematic review evidence emphasizes that most available studies focus on immediate or intermediate outcomes, with limited data on longer-term pain, disability, and quality-of-life outcomes [5]. Likewise, broader comparative evidence suggests that opioid-class therapies are associated with higher risks of adverse events and discontinuation, even when some patients experience symptom benefit [2,4]. In routine OA care, where treatment pathways frequently involve switching, combination therapy, and discontinuation over time, longer follow-up may be especially informative [1]. Short-term findings in early knee OA also suggest that outcomes such as sleep and patient-reported function may capture clinically relevant benefit not fully reflected by pain scores alone [6].

Accordingly, the present study was designed to evaluate extended outcomes associated with fixed-dose tramadol/paracetamol therapy in adults with symptomatic knee OA. Building on prior short-term observational findings in the same clinical setting [6], and considering the broader evidence on both the potential benefits and limitations of tramadol/paracetamol in OA [1,2,4,5], this study aimed to provide clinically relevant follow-up data that better reflect routine OA pain management. The primary aim was to assess changes in pain intensity using the Numeric Rating Scale (NRS). Secondary aims were to evaluate symptom burden and function using the Western Ontario and McMaster Universities Osteoarthritis Index (WOMAC), assess sleep quality using the Pittsburgh Sleep Quality Index (PSQI), and describe treatment safety and persistence, including adverse events and discontinuation. Given the retrospective uncontrolled design, the study was intended to describe exploratory longitudinal real-world outcomes rather than provide comparative evidence of treatment effectiveness.

## 2. Materials and Methods

### 2.1. Study Design and Setting

This study was designed as a single-center retrospective observational extension study based on routine outpatient clinical records of adults with symptomatic knee osteoarthritis (KOA) treated at a single clinical center. The present manuscript extends our previously published short-term retrospective report by analyzing the same clinical cohort beyond the initial 15-day observation window. The novel contribution of the current study is the inclusion of 3-month and 6-month follow-up outcome data, allowing description of the durability of symptom change and treatment tolerability over time in routine care. No intervention was assigned for research purposes; all treatment decisions were made by the treating clinician as part of routine clinical practice.

The study was conducted in accordance with the principles of the Declaration of Helsinki. Because this was a retrospective analysis of anonymized routine-care clinical records, the requirement for individual written informed consent was waived by the ethics committee. All data were handled confidentially and analyzed in anonymized form. The manuscript was prepared in accordance with the Strengthening the Reporting of Observational Studies in Epidemiology reporting principles for observational studies, where applicable.

### 2.2. Participants

Eligible participants were adults (≥18 years) with symptomatic knee osteoarthritis (KOA) managed in routine outpatient care. The analytic cohort was fixed and consisted of the same 30 eligible patients included in the previously reported short-term retrospective analysis; the present study extended follow-up in this cohort to 3 and 6 months rather than enrolling additional patients. To reduce clinical heterogeneity, only patients with knee OA and radiographic Kellgren-Lawrence grade I-II documented in the medical record were included in the analysis. Patients were included if they had symptomatic knee osteoarthritis, were prescribed oral tramadol/paracetamol in routine clinical care, and had baseline clinical data suitable for longitudinal comparison. The study describes real-world prescribing and follow-up as documented in the medical records; no treatment was assigned or modified for research purposes. Symptom duration was recorded using the date of first symptom appearance and the date of treatment initiation, allowing quantification of chronicity. Exclusion criteria were based on standard clinical considerations for tramadol/paracetamol therapy and included contraindications to opioids or paracetamol, known hypersensitivity to study medications, severe hepatic impairment, uncontrolled respiratory depression, and clinical conditions requiring alternative pain management strategies. In addition, patients who had received recent intra-articular injections or major changes in analgesic regimens immediately prior to enrollment were considered unsuitable when such interventions could confound short-term outcomes. Demographic and clinical characteristics, including age, sex, body mass index (BMI), cardiometabolic comorbidities, smoking status, and depression diagnosis, were recorded to characterize the baseline cohort and support interpretation of treatment response in a real-world context. Patients with other primary pain syndromes, inflammatory arthritides, or non-knee musculoskeletal conditions judged to be the dominant cause of pain or functional limitation were not included.

### 2.3. Treatment Protocol and Exposure Assessment

All participants initiated oral fixed-dose tramadol/paracetamol at a daily dose of 75/650 mg/day. Dose adjustment was permitted in routine care at the treating clinician’s discretion in the event of inadequate analgesia or tolerability concerns. Over follow-up, treatment exposure was characterized from the medical records by documenting maintenance of the starting regimen, any dose escalation or dose reduction, discontinuation, and the reason for change when available. Concomitant analgesic therapies and non-pharmacological interventions, including NSAID use and rehabilitation/exercise measures, were extracted from the charts when documented. Adherence and rescue medication use were assessed from routine chart documentation when available; because of the retrospective design, these variables were not uniformly recorded for all patients and are therefore reported descriptively.

### 2.4. Outcome Measures

The primary effectiveness outcome was change in pain intensity measured using the NRS between baseline (T0) and follow-up timepoints (D15, M3, and M6). Secondary outcomes included changes in the WOMAC (total score and pain, stiffness, and physical function subscales) assessed at T0, D15, M3, and M6. Sleep quality was assessed using the Pittsburgh Sleep Quality Index (PSQI) at T0 and at D15, M3, and M6, where lower scores indicate improved sleep quality. Concomitant analgesic use and non-pharmacological measures were reviewed descriptively when documented in the charts; however, because these variables were not uniformly recorded, they were not treated as formal effectiveness endpoints. Across outcomes, change (Δ) scores were computed as follow-up minus baseline (e.g., D15 − T0, M3 − T0, M6 − T0), where negative values indicate clinical improvement due to reduced pain and symptom burden.

### 2.5. Safety Outcomes

Safety and tolerability were assessed from routine clinical documentation during follow-up visits and charted patient communications. Adverse events were recorded when documented by the treating clinician or reported by the patient in the medical record. Particular attention was paid to commonly recognized opioid-related adverse effects, including nausea and constipation, as well as treatment discontinuation and dose modification due to intolerance. Because this was a retrospective real-world study, protocol-mandated laboratory monitoring was not performed for research purposes; safety evaluation was therefore based on recorded clinical assessments, documented contraindication screening at treatment initiation, and treatment-emergent symptoms captured in the charts.

### 2.6. Follow-Up Timepoints

In routine clinical practice, participants initiated tramadol/paracetamol therapy at the baseline visit (T0). For this retrospective analysis, follow-up visits documented in the medical records were identified at approximately 15 days (D15), 3 months (M3), and 6 months (M6) after treatment initiation, when available. Outcomes were extracted from the clinical charts across these four timepoints (T0, D15, M3, and M6) to describe symptom trajectories and tolerability over short- and mid-term follow-up in real-world practice.

### 2.7. Statistical Analysis

All statistical analyses were performed using IBM SPSS Statistics (version 25 or later). Continuous variables were summarized as mean ± standard deviation (SD) for approximately normally distributed data and median with interquartile range (IQR) for non-normally distributed data. Categorical variables were summarized as counts and percentages. Normality was assessed using the Shapiro–Wilk test and visual inspection of distribution plots. For within-subject comparisons between baseline and follow-up timepoints, paired-samples t-tests were used for approximately normally distributed outcomes, whereas the Wilcoxon signed-rank test was applied for outcomes that violated normality assumptions. Change (Δ) variables were calculated as follow-up minus baseline (e.g., D15 − T0, M3 − T0, and M6 − T0), with negative values indicating clinical improvement due to reduced pain and symptom burden. Because of the small sample size and the exploratory retrospective design, the analysis was intended to describe within-cohort changes over time rather than to estimate causal treatment effects. Missing data were handled using complete-case analysis at each available time point, and no imputation was performed. A two-sided *p*-value of <0.05 was considered statistically significant. For each follow-up timepoint, analyses were performed using patients with evaluable paired data for the relevant outcome. The denominator for evaluable data at D15, M3, and M6 is explicitly reported in the Section 3. No imputation was performed for missing outcome data.

## 3. Results

A total of 30 patients with symptomatic knee osteoarthritis were included in this single-center retrospective observational extension study. The baseline-to-D15 analysis derives from the same fixed cohort previously reported in the authors’ short-term publication, while the present report adds 3-month and 6-month follow-up data. Complete evaluable outcome data were available for 30/30 patients at D15, 30/30 patients at M3, and 30/30 patients at M6. No imputation was performed. Treatment discontinuation was analyzed separately as a safety and tolerability outcome. Where M3 and M6 summary statistics appear numerically similar or identical, this reflects the observed small-sample distribution and rounding of summary measures rather than duplication of data. Overall, improvements were observed across pain, stiffness, physical function, and sleep outcomes from baseline through short- and mid-term follow-up.

Baseline continuous characteristics showed that participants had a mean age of 64.8 ± 9.6 years and were predominantly in the overweight range with a mean BMI of 27.3 ± 5.0 kg/m^2^ (Table 1). Symptom duration indicated a largely chronic osteoarthritis cohort, with a mean duration of 4.5 ± 3.4 years and a wide range (0.4–12.2 years), reflecting chronic pain and functional limitation prior to initiation of combination analgesic therapy (Table 1).

At baseline, the sample had a balanced sex distribution, with 15 males and 15 females. Depression was present in 30% of patients, smoking was reported by 46.7%, and cardiometabolic comorbidities were observed in 50% of the cohort, indicating a clinically complex real-world population with factors that may influence pain perception, function, and treatment response (Table 2).

Short-term outcomes revealed significant reductions in pain intensity, a significant improvement in osteoarthritis-related symptoms and function, and sleep impairment after 15 days of therapy (Table 3). Median NRS pain decreased from 6.0 (5.0–7.0) at baseline to 4.0 (2.0–5.3) at D15 (Δ −1.9 ± 2.0; *p* < 0.001), WOMAC total score decreased from 47.8 ± 12.8 at baseline to 35.6 ± 13.8 at D15 (Δ −12.2 ± 7.0; *p* < 0.001), WOMAC pain, stiffness, and physical function also improved significantly, with all paired comparisons reaching strong statistical significance (*p* < 0.001) while sleep score improved from 9.5 (7.0–15.0) to 7.0 (5.0–9.0) (Δ −3.2 ± 2.2; *p* < 0.001), indicating meaningful improvements in pain burden, functionality and sleep quality (Table 3). These findings support early symptomatic benefit and functional gains during short-term follow-up.

**Figure 1:**
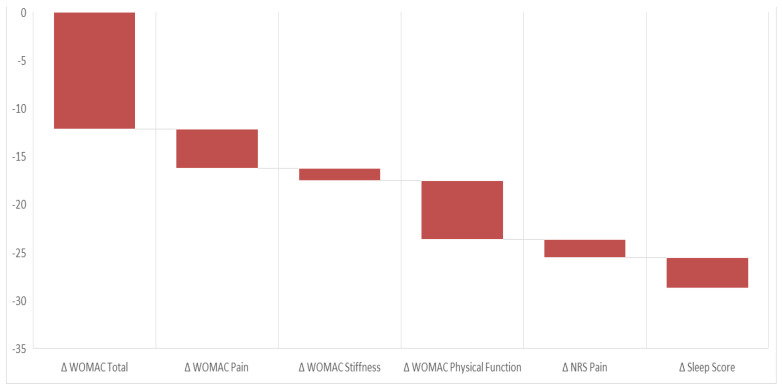
Bar chart of Mean Delta values. Mean percentage change from baseline to 15 days across patient-reported outcomes. Negative values indicate improvement due to reduction in pain, WOMAC, and sleep scores.

The bar chart summarizes the direction and magnitude of mean change (Δ) across primary and secondary endpoints, illustrating consistent negative delta values indicating improvement from baseline (Figure 1). The largest reductions were observed in WOMAC total score and WOMAC physical function, with additional clinically relevant decreases seen for WOMAC pain, NRS pain, stiffness, and sleep scores, reinforcing the overall pattern of treatment benefit across multiple patient-centered outcomes (Figure 1).

At mid-term follow-up, outcome scores remained improved compared with baseline across all assessed domains at both M3 and M6.

**Figure 2:**
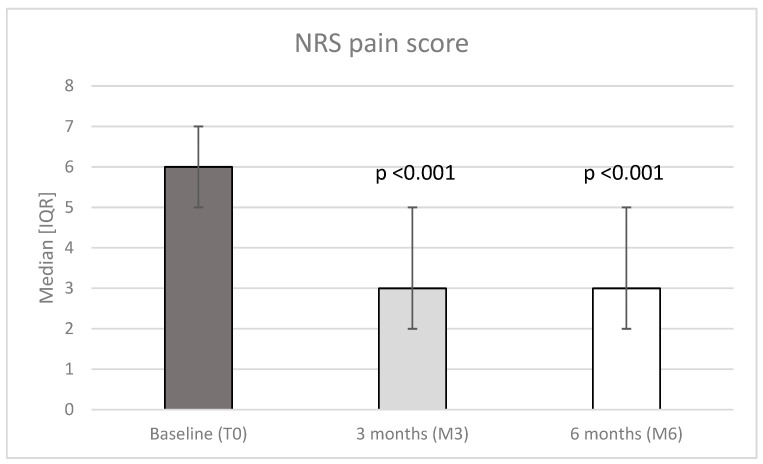
Numeric Rating Scale pain score at baseline, 3 months, and 6 months. Data are reported as median and interquartile range. *p*-values refer to paired comparisons between each follow-up timepoint and baseline.

NRS pain score decreased from a median of 6.0 (5.0–7.0) to a median of 3.0 (2.0–5.0) at both M3 and M6 (both Δ −2.4 ± 1.8; *p* < 0.001), suggesting maintenance of pain improvement at the group level over time (Figure 2).

**Figure 3:**
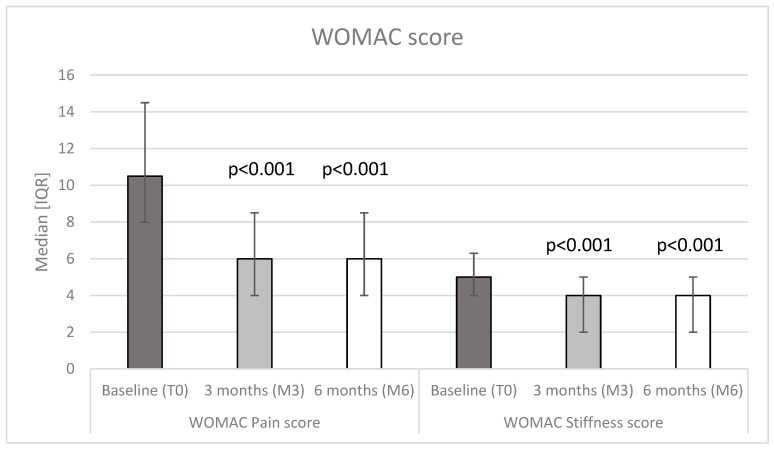
WOMAC pain and stiffness subscale scores at baseline, 3 months, and 6 months. Data are reported as median and interquartile range. *p*-values refer to paired comparisons between each follow-up timepoint and baseline.

**Figure 4:**
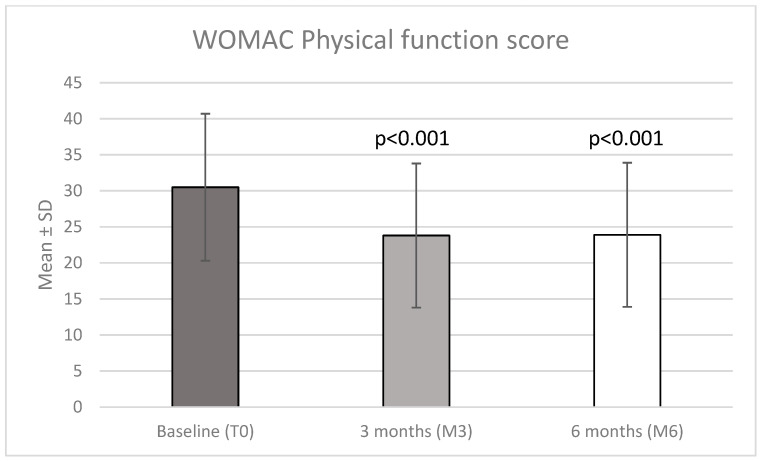
WOMAC physical function score at baseline, 3 months, and 6 months. Data are reported as mean ± standard deviation. *p*-values refer to paired comparisons between each follow-up timepoint and baseline.

WOMAC total improved from a mean of 47.8 ± 12.8 at baseline to 33.8 ± 13.6 at M3 and 33.9 ± 13.5 at M6 (Δ (M3 − T0) −14.0 ± 7.0; Δ (M6 − T0) −13.9 ± 7.0; both *p* < 0.001). Comparable sustained improvements were observed in WOMAC pain score from a median of 10.5 (8.0–14.5) at baseline to 6.0 (4.0–8.5) at both M3 and M6 (both Δ −4.9 ± 4.0; *p* < 0.001) (Figure 3); stiffness score from a median of 5.0 (4.0–6.3) at baseline to 4.0 (2.0–5.0) at both M3 and M6 (both Δ −1.4 ± 1.3; *p* < 0.001) (Figure 3), and physical function score from a mean of 30.5 ± 10.2 at baseline to 23.8 ± 10.0 at M3 and 23.9 ± 10.0 at M6 (Δ (M3 − T0) −6.7 ± 6.6, Δ (M6 − T0) −6.6 ± 6.7; both *p* < 0.001) (Figure 4).

**Figure 5:**
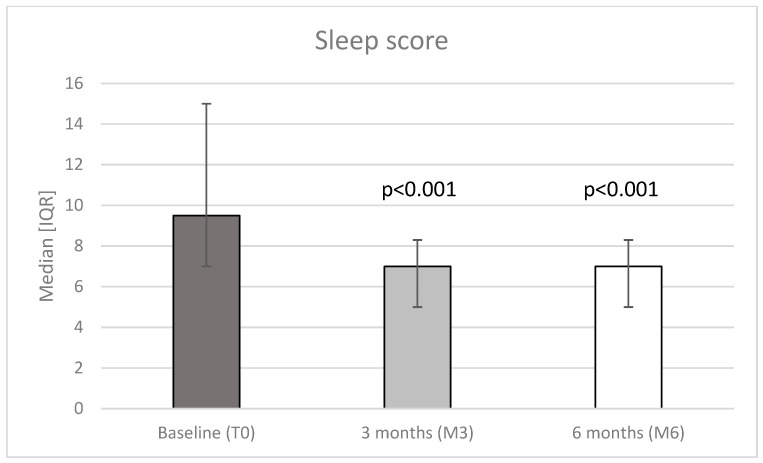
Sleep quality according to the Pittsburgh Sleep Quality Index at baseline, 3 months, and 6 months. Data are reported as median and interquartile range. *p*-values refer to paired comparisons between each follow-up timepoint and baseline.

Similarly, sleep score improved from a median of 9.5 (7.0–15.0) at baseline to 7.0 (5.0–8.3) at both M3 and M6 (both Δ −3.7 ± 2.2; *p* < 0.001) (Figure 5), supporting maintenance of sleep-score improvement through mid-term follow-up.

The tramadol/paracetamol combination therapy was generally well tolerated, with 90% of patients reporting no side effects and only 10% experiencing any adverse event (Table 4). Reported side effects were mild and infrequent, limited to nausea (6.7%) and constipation (3.3%), and treatment discontinuation occurred in only 6.7% of patients over the 6-month follow-up period, supporting an acceptable tolerability profile in this real-world osteoarthritis cohort (Table 4).

## 4. Discussion

In this small single-center retrospective observational extension study of adults with early-stage symptomatic knee osteoarthritis, fixed-dose tramadol/paracetamol was associated with improvement in pain, WOMAC outcomes, and sleep from baseline to D15, with similar group-level benefits remaining evident at M3 and M6. These results should be interpreted as exploratory within-cohort observations rather than comparative evidence of treatment effectiveness, because the study lacked a control group, included only 30 patients, and was based on retrospectively collected routine-care data. The observed median NRS change from baseline to D15 was 2 points and from baseline to M3/M6 was 3 points, which is consistent with thresholds often used to represent clinically important improvement on 0-10 numerical pain scales; however, MCID estimates for WOMAC outcomes vary across knee OA settings, so WOMAC changes in the present study should be interpreted cautiously as supportive evidence of directional patient-reported improvement rather than definitive responder analysis.

The principal novelty of the present manuscript is not the short-term response itself, which has already been reported by the authors, but the extension of follow-up through 3 and 6 months. This longer observation period addresses a clinically relevant real-world question: whether early symptomatic improvement is maintained beyond the initial treatment window in selected patients managed in routine practice.

These findings should also be interpreted within the broader context of guideline disagreement and the known trade-off between analgesic benefit and tolerability for tramadol-containing regimens. Accordingly, the present data should not be interpreted as support for routine first-line use of tramadol/paracetamol in knee osteoarthritis. Rather, they suggest that, in a selected outpatient cohort treated in routine care, short- to mid-term symptomatic improvement was observed and deserves confirmation in larger controlled studies.

The principal findings were threefold. First, the combination therapy demonstrated a fast onset of effect in NRS pain, with statistically significant improvements by D15 in WOMAC total and all WOMAC subdomains, accompanied by meaningful reductions in sleep impairment. Second, the benefits were durable, with maintained improvement at both M3 and M6 across every endpoint, indicating sustained symptom control rather than a short-lived analgesic response. Early analgesic relief may facilitate increased mobility and functional engagement, potentially reversing physical deconditioning associated with chronic pain and contributing to sustained symptom improvement. Third, the regimen showed favorable tolerability, with adverse events occurring in only 10% of participants (primarily mild nausea and constipation) and low discontinuation (6.7%). Lower AE rates compared with randomized trials may reflect real-world dose individualization, short daily exposure, and proactive patient education. This benefit–risk signal is particularly relevant for older adults with multimorbidity, a population in which NSAID exposure may be limited and opioid-class drugs raise safety concerns. Our early response pattern closely aligns with real-world observations reported by Conforti et al. [6], who evaluated the same fixed-dose tramadol/paracetamol combination (75/650 mg) in early-stage knee osteoarthritis over 15 days. Their retrospective cohort similarly demonstrated significant improvements in pain, functional outcomes (WOMAC), and sleep quality (PSQI), with adverse events reported in ~10% and no treatment discontinuation [6]. The convergence between the two studies, despite differences in design and osteoarthritis stage characterization, supports a consistent short-term symptomatic benefit of this combination in routine care. However, our longer follow-up provides added value by showing that improvements were not restricted to the acute phase; instead, outcomes remained improved at 3 and 6 months, addressing a key limitation of many short observational reports that lack durability data.

In contrast, randomized evidence and large meta-analytic syntheses often describe opioid-class analgesics as providing modest average benefits with tolerability limitations. In the BMJ network meta-analysis of knee/hip osteoarthritis pharmacotherapy, opioids as a class had a low probability of achieving clinically important pain reduction, and showed a substantially increased risk of adverse events and treatment discontinuation compared with placebo, whereas topical NSAIDs (e.g., diclofenac) demonstrated a more favorable effectiveness–safety balance [2]. Similarly, a systematic review and network meta-analysis focusing on tramadol found statistically significant but small improvements in pain (dose-dependent), with functional benefit most evident at higher dosing, and with increasing gastrointestinal/CNS adverse events and withdrawals [7]. Against this background, the magnitude and consistency of improvement observed in our cohort, together with low discontinuation, appear more favorable than what is often inferred from trial-based averages. A plausible explanation is that our study used a fixed-dose combination strategy that may achieve meaningful analgesia at lower opioid burden while allowing individualized real-world prescribing and counseling, which can influence tolerability and adherence. Our findings also help contextualize why combination therapy may outperform paracetamol alone. High-quality evidence suggests that paracetamol monotherapy provides only minimal, likely clinically unimportant improvement in osteoarthritis pain and function compared with placebo [8], a conclusion echoed by prior meta-analysis [9,10,11,12]. The robust improvements seen in our cohort, therefore, likely reflect the added analgesic contribution of tramadol and potential multi-mechanistic synergy, rather than paracetamol-driven benefit alone.

The observed trajectory, rapid improvement by D15 followed by maintenance through M3 and M6, is clinically meaningful because osteoarthritis pain management often struggles with early benefit that later wanes. Our data suggest that symptom relief was sustained rather than followed by rebound. The near-equivalent outcomes at M3 and M6 further imply a plateau effect, where patients reached a stable level of symptom control that persisted through mid-term follow-up. This pattern is consistent with earlier evidence that tramadol formulations can improve pain and function in osteoarthritis, including controlled-release strategies associated with sustained benefit and patient preference in longer extension phases [13,14,15,16]. It also aligns with evidence from sustained-release and treatment-withdrawal designs where continued tramadol exposure was associated with longer time to inadequate analgesia, although adverse events were common in many trials [17]. Collectively, these findings support the concept that tramadol-based regimens can deliver durable symptom control in selected patients, particularly when dosing strategies prioritize tolerability. In practice, the tramadol/paracetamol combination may be most useful in patients with symptomatic flares requiring fast relief, those with contraindications or poor tolerance to NSAIDs, and patients whose pain is accompanied by measurable functional limitation and sleep disruption. The latter point is particularly relevant because our cohort showed concurrent improvements in WOMAC physical function and sleep score, emphasizing multidimensional benefit. Real-life osteoarthritis management frequently involves balancing pain relief with comorbidity risk, and expert reviews highlight that although NSAIDs remain foundational, gastrointestinal and cardiovascular risks often necessitate careful selection and limitation, creating a clinical need for alternative strategies in higher-risk patients [18]. This “risk-based analgesic selection” framework supports the place of weak opioids such as tramadol in selected, severely symptomatic patients when first-line options are inadequate or unsafe [18]. Additionally, treatment decisions in real-world orthopedics increasingly reflect multimodal reasoning, where clinicians may combine agents across classes to improve outcomes while minimizing dose escalation of a single drug [19].

The improvement in sleep scores observed at D15 and sustained through M3/M6 strengthens the clinical relevance of our outcomes because sleep impairment is both a consequence and amplifier of osteoarthritis pain. Sleep outcomes are often underreported in osteoarthritis trials, yet patient-reported sleep benefit can reflect broader symptom stabilization and improved daily functioning. Prior controlled evidence has demonstrated that tramadol formulations can improve combined pain–sleep measures and health-related quality-of-life domains in painful osteoarthritis [14]. Similarly, Conforti et al. [6] documented PSQI improvement alongside WOMAC and NRS reductions in early-stage knee osteoarthritis, reinforcing sleep as a responsive patient-centered endpoint under tramadol/paracetamol therapy [6,20,21]. Our findings extend this observation by demonstrating that sleep improvement was not transient, remaining improved at mid-term follow-up. A key contribution of our study is the favorable tolerability profile: only 10% experienced mild adverse events, and discontinuation remained low (6.7%). This differs from broader opioid-class evidence where discontinuation due to adverse events is commonly elevated, and overall harms can outweigh modest benefits at the population level [2,7,22,23,24,25]. Differences in tolerability across studies may reflect several factors, including real-world dose tailoring, short-course or intermittent exposure patterns, proactive management of common opioid-related adverse effects (e.g., nausea/constipation), and patient selection. Nonetheless, caution remains essential, particularly in older adults with renal impairment or polypharmacy risk. Serious toxicity events are uncommon but have been described in high-risk settings such as postoperative patients with chronic kidney disease, highlighting the need for individualized dosing and monitoring [26]. Therefore, while our tolerability findings are reassuring, they should be interpreted within a framework that prioritizes safe prescribing and ongoing reassessment.

This study has several strengths, including mid-term follow-up, real-world clinical complexity, and assessment of multi-domain patient-reported outcomes, including pain, WOMAC domains, and sleep. The extension of follow-up through 6 months provides useful durability information that complements the previously reported short-term findings. However, the small sample size is an important limitation and has now been emphasized throughout the manuscript. The present analysis was designed as a retrospective extension of a previously described fixed cohort, rather than as a newly recruited prospective study. Therefore, adding additional patients after completion of the predefined retrospective cohort would have changed the study frame and could have introduced selection bias. For this reason, the study is presented as exploratory and hypothesis-generating, and the findings require confirmation in larger multicenter controlled cohorts.

This study also has several additional limitations. First, the sample size was small, with only 30 patients, and the study was conducted at a single center. Second, the absence of a control group prevents causal inference and does not allow the observed improvements to be separated from regression to the mean, natural symptom fluctuation, placebo effects, or concurrent clinical management. Third, because the study was retrospective, adverse events, adherence, concomitant therapies, and rescue-medication use may have been incompletely documented. Fourth, although the cohort was restricted to Kellgren–Lawrence grade I–II knee osteoarthritis, imaging was not centrally reviewed, limiting more detailed radiographic stratification. Finally, the follow-up period was limited to 6 months, so longer-term effectiveness, persistence, and safety cannot be determined. Future studies should validate these findings in larger multicenter cohorts and controlled comparative-effectiveness designs.

## 5. Conclusions

In this small, uncontrolled, single-center retrospective extension cohort of adults with early-stage symptomatic knee osteoarthritis, fixed-dose tramadol/paracetamol was associated with early improvement in pain, stiffness, physical function, and sleep, with group-level improvements remaining evident through 6 months among patients with evaluable follow-up data. Because the study was retrospective, non-comparative, and based on a limited fixed cohort, these findings should be interpreted as exploratory and hypothesis-generating rather than definitive evidence of effectiveness. Larger controlled multicenter studies with standardized exposure assessment, careful safety monitoring, and longer follow-up are required before firm clinical conclusions can be drawn.

## Figures and Tables

**Table 1:** Baseline Continuous Variables (n = 30).

Variable (Type)	Mean ± SD	Median (IQR)	Min–Max
**Age (years)**	64.8 ± 9.6	64.0 (58.8–72.3)	43–87
**BMI (kg/m^2^)**	27.3 ± 5.0	26.6 (24.0–31.2)	19.0–41.0
**Symptom duration (years)**	4.5 ± 3.4	3.9 (2.0–6.2)	0.4–12.2

**Table 2:** Baseline Categorical Variables (n = 30).

Variable (Type)	Category	n (%)
**Sex** *(Categorical)*	Male	15 (50.0)
	Female	15 (50.0)
**Depression diagnosis** *(Categorical)*	Yes	9 (30.0)
	No	21 (70.0)
**Smoking status** *(Categorical)*	Smoker	14 (46.7)
	Non-smoker	16 (53.3)
**Cardiometabolic comorbidities** *(Categorical)*	Yes	15 (50.0)
	No	15 (50.0)

**Table 3:** Pain, WOMAC, and Sleep outcomes at baseline (T0) and 15 days (D15) (n = 30).

Outcome	Baseline (T0)	15 Days (D15)	Change, Δ (D15 − T0)	*p*-Value
**NRS pain score**	6.0 (5.0–7.0)	4.0 (2.0–5.3)	−1.9 ± 2.0	<0.001
**WOMAC Total score**	47.8 ± 12.8	35.6 ± 13.8	−12.2 ± 7.0	<0.001
**WOMAC Pain score**	10.5 (8.0–14.5)	7.0 (4.8–9.5)	−4.0 ± 4.1	<0.001
**WOMAC Stiffness score**	5.0 (4.0–6.3)	4.0 (2.0–5.0)	−1.3 ± 1.3	<0.001
**WOMAC Physical function score**	30.5 ± 10.2	24.4 ± 10.3	−6.2 ± 6.8	<0.001
**Sleep score**	9.5 (7.0–15.0)	7.0 (5.0–9.0)	−3.2 ± 2.2	<0.001

Data are presented as **mean ± SD** for approximately normally distributed variables and as **median (IQR)** for non-normally distributed variables (Shapiro–Wilk test). **Δ change** was calculated as **D15 − T0** (negative values indicate improvement). Paired comparisons were performed using the **paired *t*-test** for normally distributed outcomes and the **Wilcoxon signed-rank test** for non-normally distributed outcomes. Statistical significance was set at *p* < 0.05.

**Table 4:** Safety and tolerability outcomes (n = 30).

Safety Outcome	Category	n (%)
**Side effects (reported type)**	None	27 (90.0)
	Nausea	2 (6.7)
	Constipation	1 (3.3)
**Any adverse event (AE)**	No AE	27 (90.0)
	AE present	3 (10.0)
**Treatment discontinuation**	No	28 (93.3)
	Yes	2 (6.7)

“Any adverse event (AE)” reflects the presence of any reported side effect (including nausea or constipation). Treatment discontinuation refers to the interruption of therapy during the 6-month follow-up.

## Data Availability

The data presented in this study are available from the corresponding author upon reasonable request.
